# Future prospects of Qiliqiangxin on heart failure: epigenetic regulation of regeneration

**DOI:** 10.3389/fgene.2013.00221

**Published:** 2013-10-24

**Authors:** Lichan Tao, Shutong Shen, Xinli Li

**Affiliations:** Department of Cardiology, The First Affiliated Hospital of Nanjing Medical UniversityNanjing, China

**Keywords:** heart failure, epigenetic regulation, regeneration, Qiliqiangxin, prospects

Qiliqiangxin (QL), a traditional Chinese medicine, was approved by China Food and Drug Administration for the treatment of heart failure in 2004 (Li et al., 2013). Our recent multicenter, randomized, double-blind, parallel-group, placebo-controlled study of the effects of QL capsules in patients with chronic heart failure shows that on a background of standard treatment, QL capsules further decreases the levels of plasma N-terminal pro-B-type natriuretic peptide (NT-proBNP) during 12 weeks of treatment (Li et al., 2013). However, the underlying mechanism is still unclear.

Recent progress in studying QL on improving exercise tolerance and patients' quality of life highlighted that QL may be through a variety of biological mechanisms to exert its beneficial effects. One possible mechanism may be the regulation of the balance between pro-inflammatory and anti-inflammatory cytokines in cardiomyocytes (Xiao et al., 2009). In addition, it may down-regulate the cardiac chymase signaling pathway and chymase-mediated AngII production (Zhao et al., 2012). Moreover, it may affect cardiomyocyte death and proliferation, leading to improved cardiac remodeling and cardiac function (Zou et al., 2012).

Traditionally, the adult mammalian heart is regarded as a post-mitotic organ without any regenerative capacity (Rosenzweig, 2012). Accumulating evidence shows that cardiomyocytes in adult mammals actually retain a limited ability to proliferation (Rosenzweig, 2012). Recently, a study shows that exercise induce physiological hypertrophy featured by cardiomyocyte hypertrophy and proliferation. The cardiomyocyte proliferation is mediated by the reduction of C/EBPß and a linked increased of CBP/p300-interacting transactivator with ED-rich carboxyterminal domain 4 (CITED4) (Boström et al., 2010). Interestingly, QL has been reported to decrease C/EBPß and also increase CITED4 at 4 weeks after transverse aorta constriction induced cardiac hypertrophy, remodeling and dysfunction in mice (Zou et al., 2012). The changes of C/EBPß and CITED4 are paralleled with enhanced proliferation of cardiomyocytes (Zou et al., 2012). Moreover, our recent study also proves that Huangqi (one of the major components of QL) improves cardiac function after acute myocardial infarction in mice by regulating mTORC1 signaling pathway, which also medicates cell proliferation (Wu et al., 2013).

In conclusion, we think QL may mimic a phenotype of physiological hypertrophy and induce heart regeneration, which is beneficial for heart failure.
